# Improvement of the adhesion of conductive poly(m-toluidine) onto chemically reduced-wool fabrics

**DOI:** 10.3906/kim-2002-77

**Published:** 2020-06-01

**Authors:** Meryem KALKAN ERDOĞAN, Meral KARAKIŞLA, Mehmet SAÇAK

**Affiliations:** 1 Department of Chemistry, Faculty of Science, Ankara University, Ankara Turkey

**Keywords:** Surface modification of wool, reduction pretreatment, conductive polymer, poly(m-toluidine), EMI shielding, rubbing stability

## Abstract

Wool has disulphide bonds containing-hydrophobic external keratin layers, which act as a barrier for the modification through coating with hydrophilic materials. For that reason, in this work, to ensure a dense and homogenous conductive polymer coating onto the wool, the fabrics were subjected to the reduction process in the aqueous alkaline medium containing agents that can attack these disulphide bonds. Then, one of the polyaniline derivatives, poly(mtoluidine) (PMT), was coated onto wool by in situ polymerization of m-toluidine sulphate using ammonium persulfate (APS) as an oxidant. The effects of conditions, such as the composition of reduction-bath and types of dopants were investigated, on the mass increase (%) and surface resistivity of the composite. The reduction pretreatment of wool with sodium hydrosulphide significantly improved the coating density, conductivity, and colour shade of PMT on the surface, compared to an untreated one. The coating stability of PMT/wool composite was examined by rubbing test and detergent washing, through surface resistivity measurements. The changes in structural and surface properties of wool fabrics were determined with ATR-FTIR, contact angle, and optical microscopic techniques, respectively. The performance of PMT/wool composite was also examined in the electromagnetic shielding effectiveness (EMSE) measurements within 30 MHz-3 GHz.

## 1. Introduction

Wool is one the most preferred natural polymer that has been used as a textile material, due to its unique properties such as keeping warm, having secure maintenance/handling, and biocompatible. The functional groups present in the polypeptide structure of wool also enable to modify their surfaces with different chemicals for fulfilling the requirements of different application fields, such as bio-textiles, adsorption, and catalytic activity [1,2]. However, the disulphide linkages containing external keratin layers limit the usage of wool for the modification of its surface, such as coating with a hydrophilic chemical. For that reason, many attempts have been made for lowering the disulphide crosslinking density of wool keratin through wet chemical approaches such as grafting of vinyl monomers [3–5], modification with chitosan [6,7], ionic-liquid treatment [8], and extraction by chemical reduction [9–12] in the literature. Among them, thanks to providing irreversible disulphide cleavage of keratin with high reaction yields [10], chemical reduction methods are found promising by the researchers. In the method, the disulphide link of keratin can be converted into oxidized another disulphide form (reconstruction) [10,13] or disrupted to give SH groups, through chemical reducing agents that are capable of attacking disulphide links containing S atoms such as mercaptoethanol, L-cysteine, thioglycolic acid (TGA), and sodium hydrosulphide hydrate (NaHS.xH_2_O) under controlled pH in the presence or absence of urea [8–11,14,15]. In this work, we preferred the use of TGA and NaHS as organic and inorganic reducing agents for the cleavage of disulphides, respectively.

The present work aims homogenously and densely modification of the hydrophobic wool surface, by a hydrophilic conductive polymer after the application of a chemical reduction pretreatment. With the help of this, less pronounced polyaniline derivative, PMT, could be coated in high yields onto reduced-wool fabrics through the in situ oxidative polymerization method. Although there have been studies reporting the preparation of other conductive polymers/wool in the literature [16–21], to our knowledge, no study has been present related to the employment of chemically reduced-wool for the conductive polymer coating. The published studies investigated only the pretreatment of wool with chemical methods [16,19–21]. Only in a few studies, the atmospheric plasma treatment [16] and oxidative treatment (shrink-proofing, bleaching, or depigmentation) [22] were reported for the improvement of polypyrrole adhesion onto wool. Our presented study can be found differentiated from the other works in terms of many points. For example, in this study, we used a relatively more cost-effective method and apparatus compared to plasma treatment and employed environmental-friendly chemicals that might have minimal harmful effects on nature such as TGA, NaHS, and urea in the aqueous alkaline media.

Moreover, we investigated the effects of these reducing agent types on the conductive mass increase (%) and surface electrical resistivity. We also monitored the coating stability of the composites with environmental conditions such as rubbing and washing tests. The prepared composite was also examined in the electromagnetic interference (EMI) shielding application in the near field (radio and low microwave) frequency range between 30 MHz–3GHz.

## 2. Materials and methods

### 2.1. Materials

Wool fabrics (Yünsa Wool Industry and Trade Co., Tekirdağ, Turkey) were washed before use in 4g/L of aqueous ECE (B) reference detergent (James Heal) solution at 40 °C for 1h. The fabrics were then dried in a vacuum oven at 50 °C until constant weight. The chemicals that were written in the process sequence, including NaHS.xH_2_O (90%), urea (99.5%), thiourea (99%), thioglycolic acid (TGA) (80%), NaOH (98%), HCl (37%), ethyl alcohol (96.5%), m-toluidine (99%), m-toluidine sulphate (99%), ammonium persulfate (APS) (98%), ((NH_4_)_2_S_2_O_8_), H_2_SO_4_ (96.5%), and methanol (99.8%) were obtained from Sigma-Aldrich and used without further purification.

### 2.2. Methods

#### 2.2.1. The reduction of keratin disulphide linkages of wool fabrics

The reduction of keratin disulphide (-S-S-) linkages of wool fabrics were performed using either inorganic (NaHS) or organic (TGA) ingredients according to a report published in the literature [9]. The components of the reduction baths are listed in Table 1. In the reduction process with inorganic chemicals, 1 g wool fabric was immersed into 2.0 M 50 mL aqueous urea solution containing 2-neck round bottom glass and stirred at 35 °C under N_2_ atmosphere. Then, 1 g NaHS was introduced to the medium, and the pH of the solution was adjusted to 10.5 with 2.0 M NaOH. The reduction reaction was continued for 24 h at the same temperature. Another inorganic reduction procedure was also performed similarly, using 50 mL distilled water instead of 2.0 M 50 mL aqueous urea solution as the medium for comparison. In the other reduction process with organic chemicals, 1 g wool fabric sample was immersed into 1.5 M 50 mL aqueous thiourea solution, and 0.625 mL TGA was then added to the solution by stirring at 35 °C under N2 atmosphere. After the adjustment of the pH to 10.5 with 2 M NaOH, the reduction proceeded for 24 h. Another organic reduction procedure was also applied to the wool fabrics using 50 mL distilled water instead of 1.5 M 50 mL aqueous thiourea solution for comparison. At the end of the 24 h, the fabric samples were separated from the aqueous solutions (for both inorganic and organic media) by filtration, washed thoroughly with water, acidified using 6.0 M HCl until reaching pH 4–5 and rinsed with ethyl alcohol. The aqueous fractions were also acidified with 6.0 M HCl, and the reduced keratinous substances were precipitated. Finally, the precipitated contents and wool fabrics were dried at 50 °C under vacuum until constant weight. The weight loss values (%) of the wool fabrics after reduction processes were calculated gravimetrically by taking into account the weights of wool fabrics before and after reduction processes.

**Table 1:** The bath ratios used in the inorganic and organic chemicals containing reduction processes of wool fabrics.

Run	Inorganic (NaHS.xH_2_O)	Organic (TGA)
1	1 g wool/1 g NaHS/50 mL water	1 g wool/0.5 g TGA/50 mL water
2	1 g wool/1 g NaHS/50 mL 2.0 M urea	1 g wool/0.5 g TGA/50 mL 1.5 M thiourea

#### 2.2.2. Preparation of PMT coated-wool fabric composites

A wool fabric sample (1 cm ×1 cm sizes) was laid on a glass tube and 0.1 M (0.1561 g) 2.5 mL of m-toluidine sulphate solution, prepared in the determined aqueous acid solution (1.0 N HCl or 1.0 N H_2_SO_4_), was dropped onto the fabric. After cooling the solution down to 5–10 °C, the polymerization was initiated by the drop by drop addition of 0.05 M APS solution prepared in the same acid solutions [23] and continued at this temperature range for 24 h. At the end of 24 h, the PMT/wool composite was removed from medium and thoroughly washed in distilled water and methyl alcohol, to remove the residual reactants. Subsequently, the composite was redoped in the same acid solution used in the polymerization and dried in an oven at 50 °C under vacuum. The mass increase (%) of the composite was determined gravimetrically, by dividing the difference of the initial and final dry weights of the wool fabrics to the initial fabric weight.

**Figure 1:**
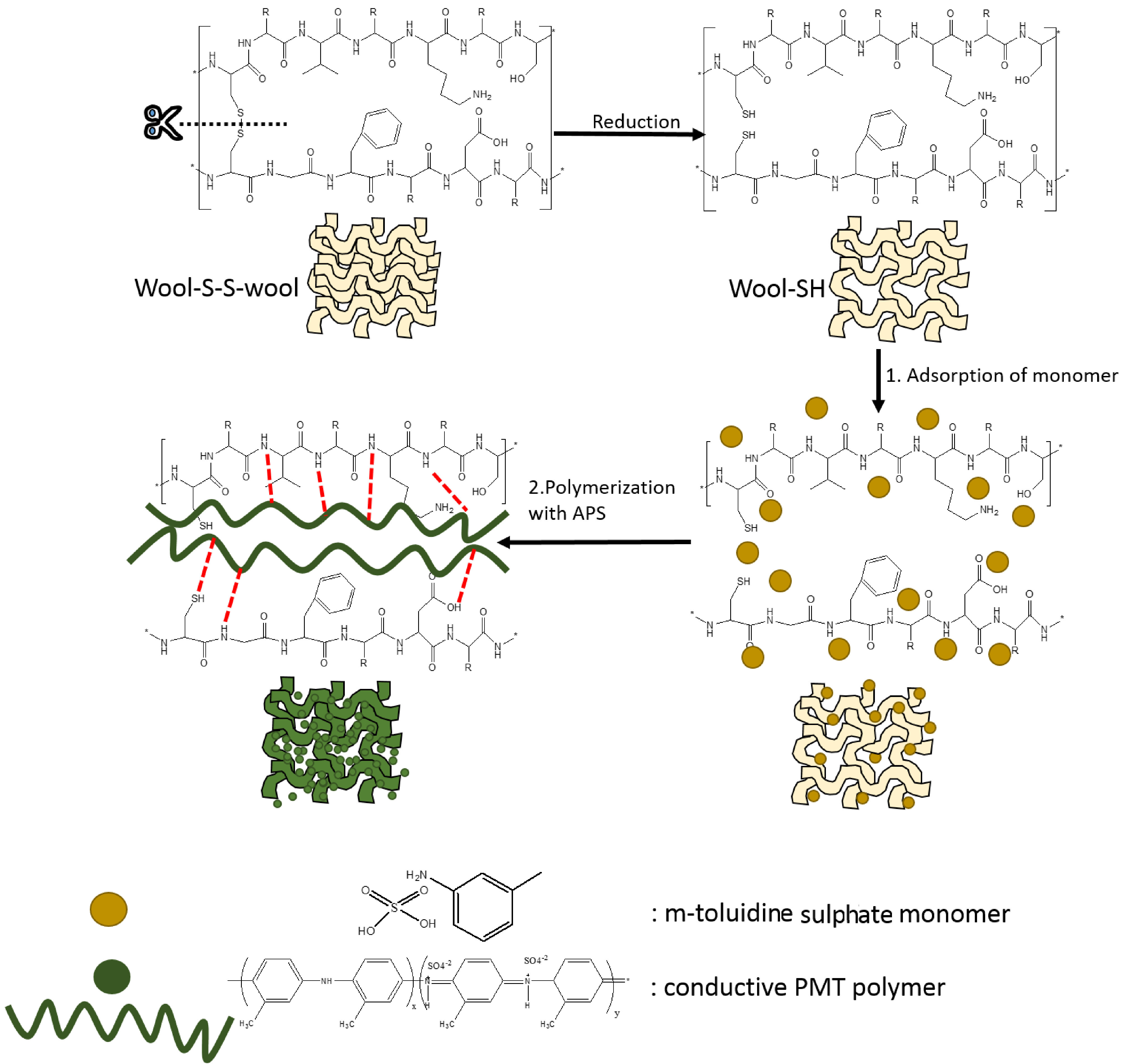
The preparation steps of the composite and possible interactions between PMT polymer and the reduced-wool surface.

The preparation steps of the composite and possible interactions between wool [1] and PMT are illustrated in Figure 1.

### 2.3. Characterization

The surface resistivity (in Ω/cm^2^) values of the composite samples were measured by 2 probe methods, using Thurlby 1503 digital multimetre for the samples whose surface resistivity is lower than 3.2 ×10^7^ Ω/cm^2^ and Uni-T UT70A for those of lower than 2 ×10^9^ Ω/cm^2^. The average of 10 different measurements taken from different faces of a sample was given as the surface resistivity result. The ATR-FTIR spectra of the samples were recorded with Perkin Elmer Spectra 100 FTIR spectrometre. The optical microscope images were taken with the Optika Microscope equipped with a camera. The contact angle-wetting time measurements were performed using the Attension Theta Lite Optic Contact Angle instrument with the sessile drop method.

### 2.4. Stability tests

#### 2.4.1. Rubbing stability

The stability of the PMT coated-wool samples against rubbing was determined according to the “AATCC Test Method 8-2005 Colourfastness to Crocking: AATCC Crockmetre Method”. The principle of the method includes the rubbing of a 5 cm ×13 cm in sizes of PMT-coated fabric specimen (in the dry state) with a white test cloth (in 1 cm ×1.7 cm sizes, white bleached cotton) in the long dimension of the specimen for 10 times (with a speed of 1 rubbing/s). The change in the surface resistivity of the specimen was monitored after the rubbing process.

The colour transferred to the white test fabric was determined by the colour difference values (ΔE), obtained from C.I.E. L a b colour coordinates through a professional image processing Adobe Photoshop CC 2019 software. CIE L a b colour coordinates are 3-dimensional colour space system and defined by Commission Internationale de l’Eclairage. ΔE value is characterized by the distance between 2 colour coordinates in the colour plane and can be calculated according to Equation (1):

(1)ΔE=[(ΔL)2+(Δa)2+(Δb)2]1/2

**Figure 2:**
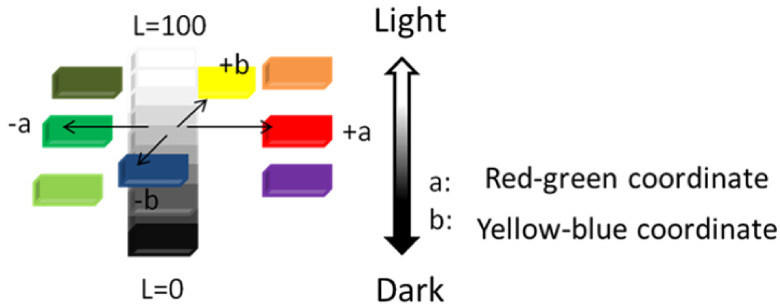
The L a b coordinate system and their corresponding colours.

A high ΔE value corresponds to a higher distance between reference and specimen colour planes. The L a b coordinate system and their corresponding colours are given in Figure 2.

#### 2.4.2. Washing stability

The durability of the surface resistivity of the PMT/wool composites against detergent washing was determined according to our previous work [23]. Briefly, a composite sample in 1 cm ×1 cm size was stirred into 4 g/L ECE (B) reference detergent containing solution at 20 °C for 30 min. Then, the composite was rinsed well with distilled water, laid on glass, and dried in a vacuum oven at 50 °C for 24 h. After conditioning in a chamber at constant moisture, the surface resistivity of the composite was measured. This procedure, including detergent washing-drying-surface resistivity measurement, was repeated a few times.

### 2.5. EMSE measurements

The EMSE measurements were carried out using a test fixture (Electro-Metrics EM-2107A) connected to a Network Analyzer (Agilent E5061B Vector) in the frequency range of 30 MHz to 3 GHz. The instrument calculates the EMSE value of a composite in decibel (dB), considering Equation (2) [24].

(2)EMSE=10logPTPI

The *P_T_* and *P_I_* values indicate the power strengths of transmitted and incident electromagnetic waves, respectively. The transmittance (T), showing the transmitted electromagnetic wave from the composite (the failure of the composite), and reflection (Re) loss attenuated through the reflection from the composite surface can be calculated according to Equation (3) using the S parameters:

(3)T=|EtEi|2=|S21orS21|2andRe=|ErEi|2=|S11orS22|2

The values of E_t_, E_i_, and E_r_ are the transmitted, incident, and reflected electrical fields strengths, respectively. S_12_ (or S_21_) and S_11_ (or S_22_) values correspond to the attenuation on transmittance and the reflection measurements, respectively. Since the S parameter values are negative (–) values, in the displaying of the EMSE result of the composite, the absolute IS_12_I (or IS_21_I) values were used.

## 3. Results and discussion

**Figure 3:**
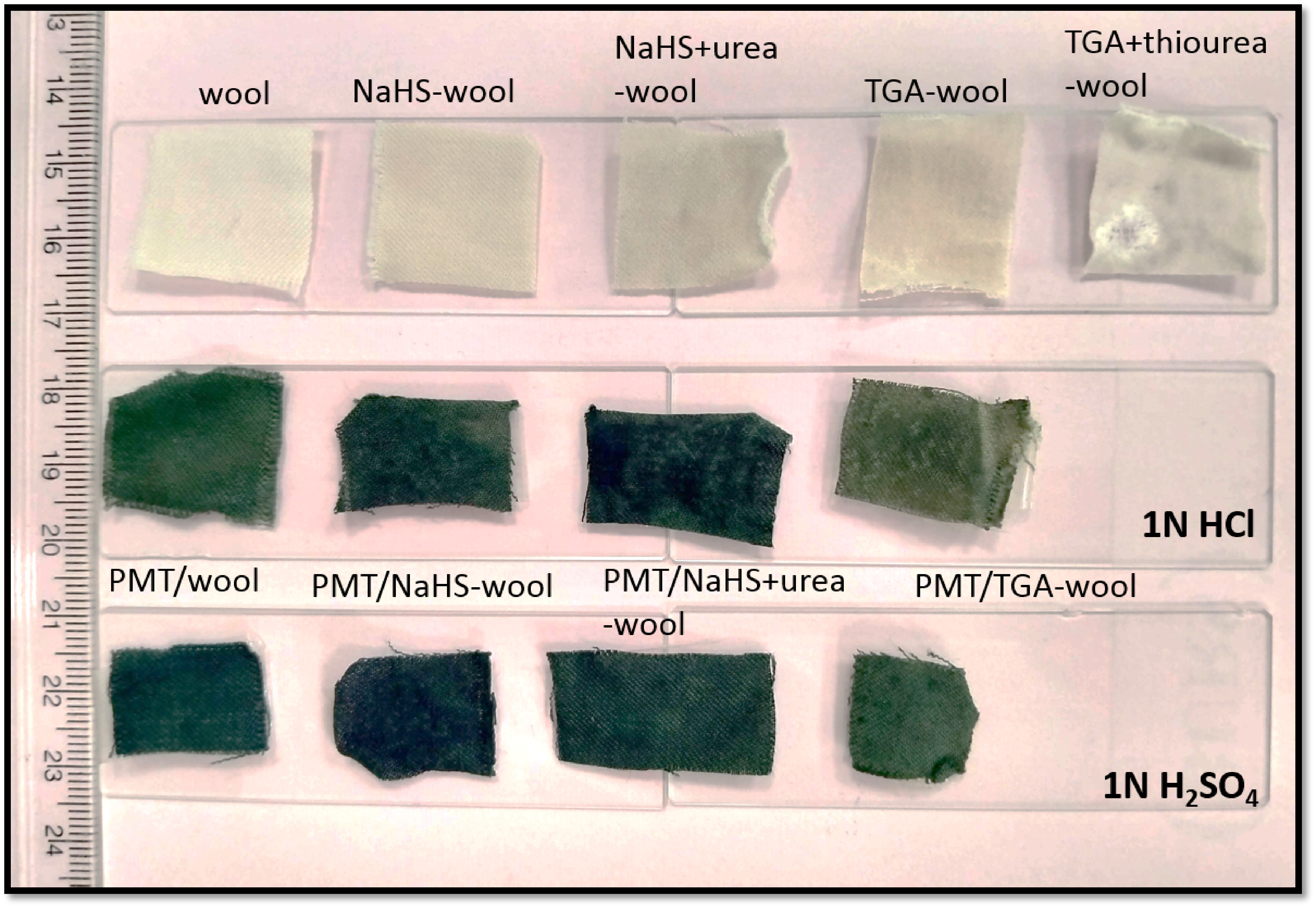
Photographic images of the pretreated and PMT coated-wool samples.

In the first attempts for the preparation of a conductive PMT coated wool fabric composite, an untreated wool fabric was directly used in the experiments. Although a measurable increase was obtained (2.5%) in the weight of the fabric after coating with PMT, the density and colour shade of the PMT coat on the fabric surface became remarkably low. The appearance of the composite gave the impression of PMT particles were powder-like spread on the surface of wool (Figure 3), due to the presence of cuticle scales containing disulphide (-S-S-) cross-linkages that make fabric inert against its modification [25]. For that reason, to form -SH groups that tend to adsorb cationic substances such as conjugated monomers, and thus, to enable the modification of the surface with chemical methods such as coating, the wool fabrics were subjected to the reduction process in the aqueous alkaline medium containing reducing agents with sulphide atoms [9,11,13,14]. For that purpose,2 different common reducing agents, including inorganic (NaHS) and organic (TGA) were preferred for the pretreatment of wool surface in the presence or absence of urea/thiourea. Accordingly, the weight loss (%) and observational change results in the fabric handling properties are summarized in Table 2. In the first view of the results, it can be seen that the weight loss (%) values of the wool fabrics pretreated in the urea and thiourea containing reducing baths were remarkably higher than those of the absent ones. It can also be noticed that the weight loss values obtained in the NaHS and NaHS/urea containing baths relatively lower than that of the TGA employed-media, suggesting that the TGA may play a more dominant role in the reducing of wool, as compatible with the literature [9,11]. This finding can also be evidenced by the observational change results of the reducing baths (also can be seen from Figure 3) since the fabric took more deteriorated form after TGA reduction pretreatment, especially that in TGA/thiourea medium. Additively, it was apparent from the images that the weaving tightness of the fabrics, in other words, the spaces between the fibres of wool woven expanded, and this observation was more noticeable for urea/thiourea treated samples. Consequently, the PMT coating of wool experiments was further continued in NaHS, NaHS-urea, and TGA media, where the wool fabric form was preserved.

**Table 2:** The weight loss (%) and observational change results of wool fabrics pretreated in different reducing baths.

Reducing agents	Weight loss (%) of wool	Deterioration in the fabric handling
NaHS	12.9	-+
NaHS+urea	23.8	+
TGA	18.6	++
TGA+thiourea	32.7	+ + +

-+: slightly, +: moderate, ++: remarkable, + + +: very remarkable/losing fabric form

After the determination of the reducing bath reagents, the conductive PMT coating process was performed in the presence of these pretreated fabrics using either m-toluidine (liquid form) or m-toluidine sulphate (solid) as the monomer, in a 1.0 N HCl medium that is frequently preferred in the preparation of highly conductive polyanilines [23]. When m-toluidine was employed in the polymerization, it was observed that the PMT polymer was coated regionally on the surface of the fabrics and the mass increase (%) of the composite became relatively low (average of virgin and NaHS-treated wool: 1.0%), due to the insufficient penetration of the monomer. The surface resistivity of the composite was also remarkably high (average 997 kΩ/cm^2^). When the m-toluidine sulphate solution was used in the experiment, the surface of the pretreated wool fabrics was homogeneously/continuously covered with PMT, with a relatively high mass increase, even for the untreated wool fabric (Table 3). Accordingly, the m-toluidine sulphate was selected as a monomer in further experiments. Moreover, although relatively the lowest surface resistivity of the composite was obtained with the use of NaHSwool fabric as 95 kΩ/cm^2^, the values of the other composites pretreated with NaHS-urea and TGA were close to each other. For that reason, all the pretreated fabrics were simultaneously used in the preparation of PMT/wool composites.

**Table 3:** The change in mass increase (%) and surface resistivity of PMT/wool composites with the reducing agents used in the pretreatment of wool.

Run	Reducing agent	Surface Resistivity (kΩ/cm^2^)	Mass increase (%)
0	None*	1370	0.8 ±0.2
	NaHS*	623	1.4 ±0.4
1	None	218	2.5 ±1.5
2	NaHS	95	3.0 ±0.1
3	NaHS+urea	111	4.0 ±1.7
4	TGA	107	4.1 ±1.0

*0.10 M m-toluidine solution was used as a monomer [m-toluidine sulphate]: 0.10 M and [APS]: 0.05 M prepared in 1.0 N aqueous HCl solution.

**Figure 4:**
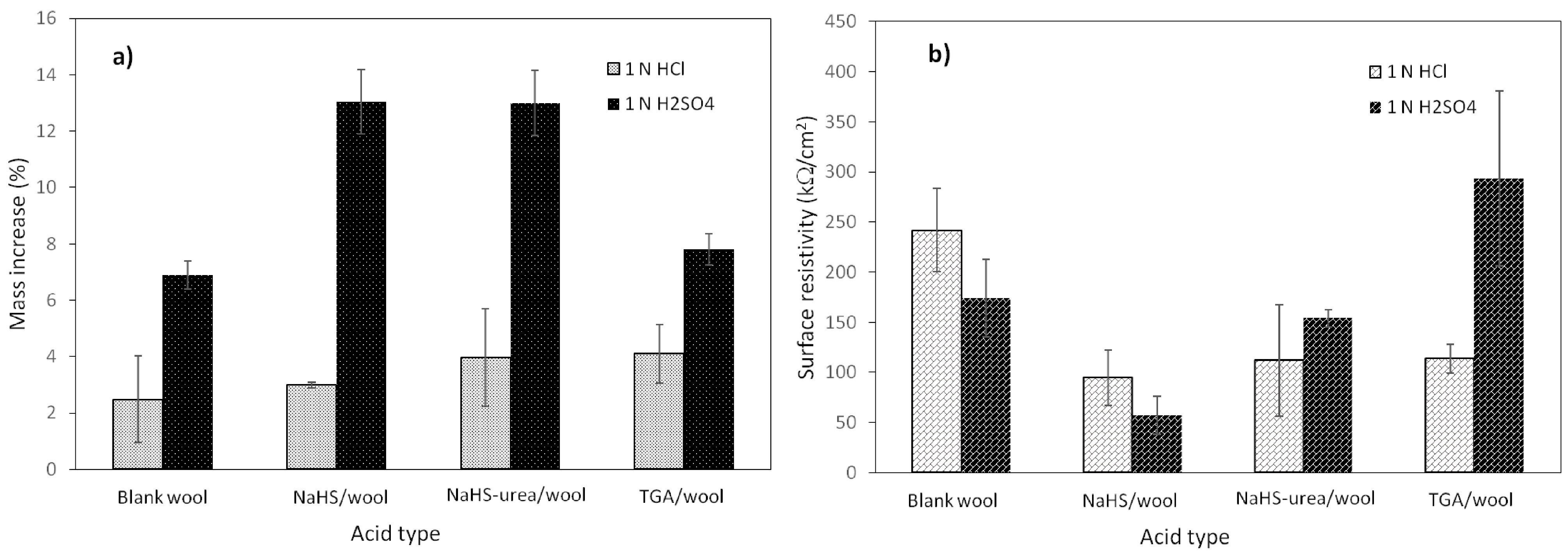
The changes in the mass increase (%) and surface resistivity of various PMT coated-wool composites with the type of acid used in the polymerization ([m-toluidine sulphate]: 0.10 M and [APS]: 0.05 M).

To determine the effect of the dopant acid type on the mass increase (%) and surface resistivity of composite fabrics, the polymerization reactions were repeated using either aqueous H_2_SO_4_ or HCl solutions that have different counterions at the constant equivalent concentration (1N). The results, including the changes in the mass increase (%) and surface resistivity of the wool fabrics, are displayed in Figures 4a and 4b, respectively. As seen from Figure 4a, the mass increase (%) of the composites prepared in 1.0 N HCl medium was almost close to each other and took relatively low values when compared to those of H_2_SO_4_. In the 1.0 N H_2_SO_4_ medium, it was observed that the mass increases (%) of the composites varied depending on the reducing agents used in the pretreatment, and the highest mass increase (%) could be obtained with the NaHS pretreated-wool fabrics as 13.0%. Although the mass increase of virgin wool took the lowest value in 1.0 N HCl medium, in 1.0 N H_2_SO_4_, its value remarkably increased and became closer to that of TGA pretreated-wool fabric.

When the changes in the surface resistivity of the composites are examined (Figure 4b), the trend of the fabrics that were pretreated with different reducing agents behaved differently. Interestingly, although relatively the highest mass increase (%) was obtained with TGA pretreated-fabric in 1.0 N HCl medium, the NaHS pretreated-wool had the lowest surface resistivity in both of the acidic media, especially that in 1.0 N H_2_SO_4_ (57 kΩ/cm^2^), suggesting that the surface resistivity of the PMT/NaHS-wool almost identical in HCl and H_2_SO_4_. To conclude, the composite with the highest mass increase (%) and lowest surface resistivity could be prepared in 1.0 N H_2_SO_4_ medium.

### 3.1. ATR-FTIR spectra

**Figure 5:**
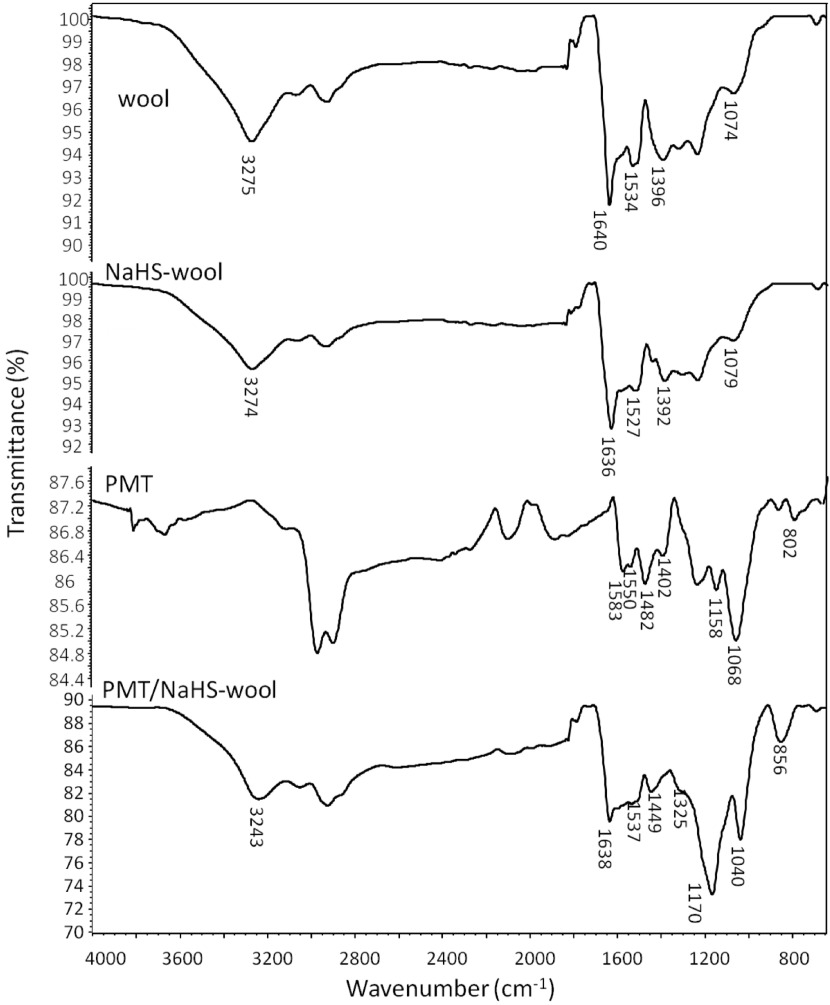
ATR-FTIR spectra of untreated wool, NaHS-wool, PMT polymer, and PMT/NaHS-wool.

The ATR-FTIR spectra of the samples are comparatively presented in Figure 5. As seen from the wool spectrum, the bands located around 3275, 1640, and 1534 cm^-1^ are attributed to the stretching vibrations of peptide bonds, including N-H, C=O (amide-I), and bending vibration of N-H (amide-II) [12,15]. The other bands at 1396 and 1074 cm^-1^ are also assigned to the stretching vibrations of the axial deformation of C=O and S-O groups, respectively [1]. After the reduction of wool keratin with NaHS, the bands corresponding to the defined peptide bonds also detected with the slight shifts to the lower wavenumbers. This observation may suggest that the protein structure of the wool was not significantly affected by the NaHS reduction process, as similarly observed with the literature [15]. When the shifts occurred in the bands of amide-I are considered, it can be said that the secondary structure of the wool proteins may have been changed because in the studies reporting the extraction of the wool keratin [10,27] the changes in the position of the amide I band in the FTIR spectra were attributed to a change from α-helix (ca~1650 cm^-1^) to β -sheet structure (ca. ~1630 cm^-1^).

In the spectrum of the pure PMT polymer, while the bands at 1583 and 1550 cm^-1^ correspond to the C=C stretching and C-H bending modes of quinoid rings, respectively, the bands at 1482 and 1402 cm^-1^ can be assigned to the C=N antisymmetric stretching and C-H bending modes of benzenoid rings [28,29]. The band centred at 1158 cm^-1^ can be related to the stretching vibration of protonated-polyaniline units (quinoid=NH+−benzenoid or benzenoid−NH+−benzenoid) in the literature [30,31]. The strong band positioned around 1068 cm^-1^ can be attributed to the antisymmetric stretching mode of SO_4_^2-^ ions that contribute to the PMT chains as dopant counterions [28,32,33] and the presence of this band was reported to involve in the crosslinking of polyaniline chains through hydrogen bonding [28]. Finally, the band at 802 cm^-1^ can be assigned to the out of plane deformation of 1,4-disubstituted benzenoid rings of PMT that is reported to be observed ca.~820 cm^-1^ for unsubstituted polyanilines in the literature [30,31].

In the composite spectrum, it was observed that the bands belonging to NaHS-wool fabric were preserved. Due to the superimposition with those of the PMT polymer, some bands were also expanded with shifts. For example, the bands of PMT around 1583–1550, 1482–1402, and 1158 cm^-1^ were superimposed with those of wool and observed at 1537, 1449, and 1170 cm^-1^ , respectively. The shifts of the bands corresponding to the SO_4_^2-^ ions and disubstituted benzenoid rings of PMT at 1040 cm^-1^ and 856 cm^-1^ are thought that a strong interaction such as hydrogen bonding may have occurred between PMT chains and wool backbone.

### 3.2. Contact angle-wetting time measurements

**Figure 6:**
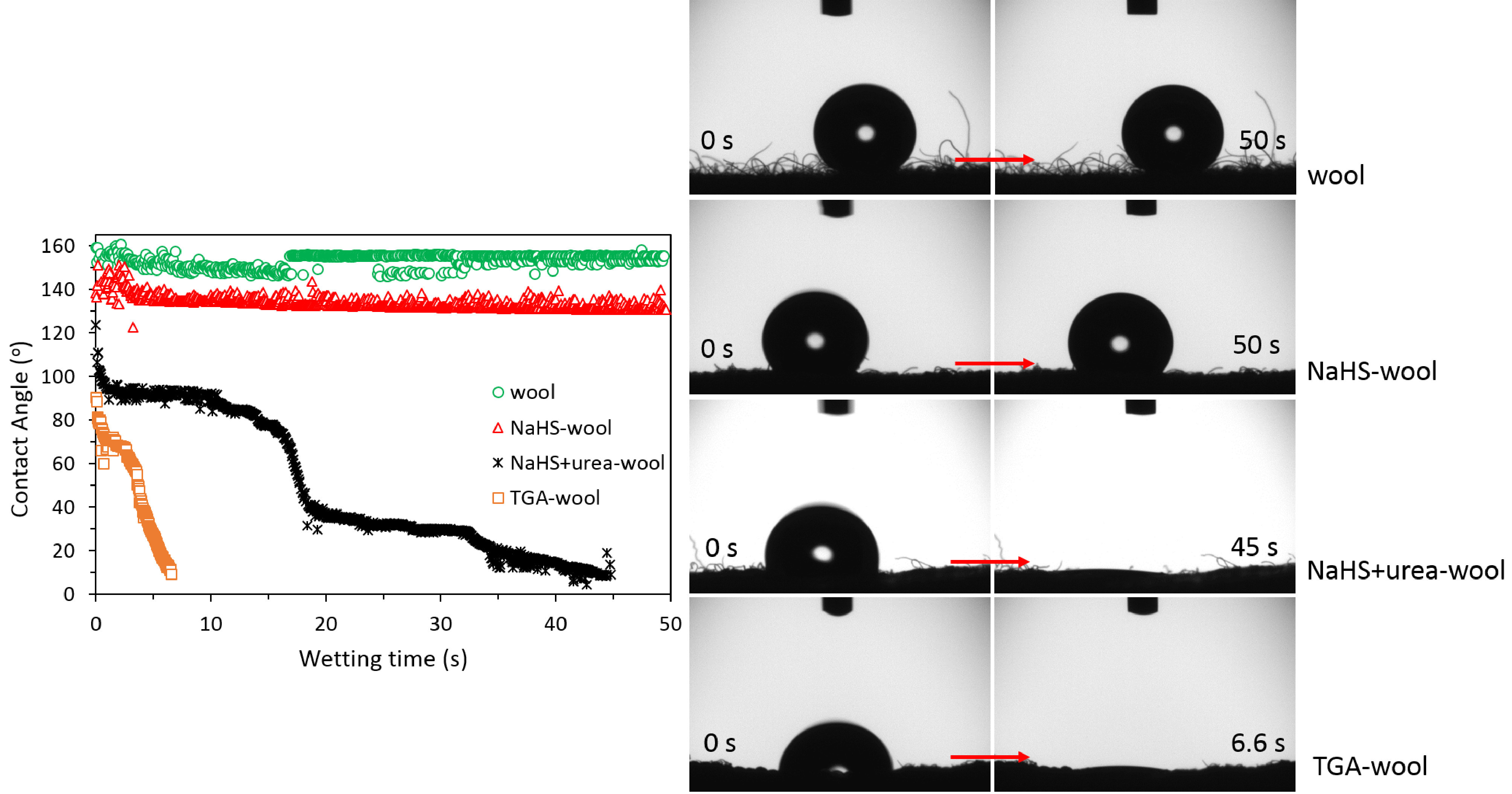
Contact angle-wetting time values of wool, NaHS-wool, NaHS+urea-wool, and TGA-wool fabrics.

**Figure 7:**
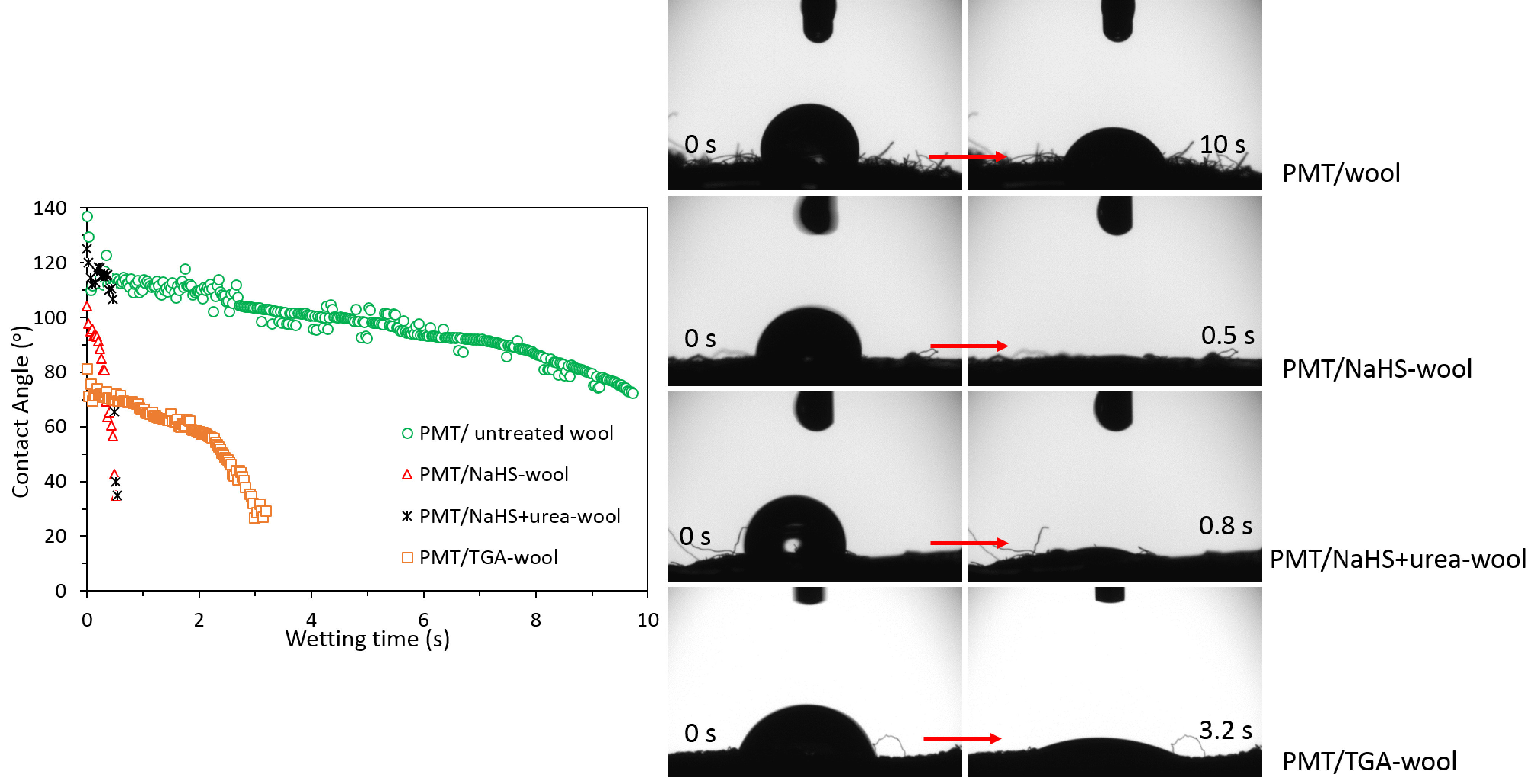
Contact angle-wetting time values of PMT/wool, PMT/NaHS-wool, PMT/NaHS+urea-wool, and PMT/TGA-wool fabrics.

The changes in the hydrophilic character of the wool fabric after reduction pretreatments and PMT coating were evaluated through water contact angle and wetting time measurements, and the results are given in succession with Figure 6 and Figure 7.

As seen from Figure 6, the water contact angle of untreated wool fabric changed around 150°, indicating the hydrophobic/nonwetting character of the wool. After the treatment of wool fabrics with reducing agents, it was observed that the contact angle/wetting time values of the fabrics decreased in the order of weight losses/deterioration of the fabrics: NaHS-wool (134°, >the 50s), NaHS+urea-wool (111°, 45s), and TGA-wool (90°, 6.6s). This finding indicates that the hydrophilicity of the fabrics improved with the usage of a harsh reducing agent (TGA), and this could only be ensured through the removal of keratin layers from the surface, rather than the formation of SH groups because the SH bonds do not provide a hydrophilic character similar to the OH bonds. This case can also be evidenced by the PMT coating homogeneity of the composites since the composite with the highest mass increase (%) was obtained when a NaHS-wool fabric was employed. By taking into account the mass increase (%) and contact angle relations of the composites, it can be postulated that the NaHS treatment may have provided the highest SH content (with the lowest hydrophilicity), leading to the most homogenous/dense PMT coating. The dense PMT coat onto the NaHS-wool surface can also be easily selected from the optical microscope images of the composites that are comparatively given with PMT/wool and PMT/NaHS-wool, in supplementary Figure 1S.

When the contact angle wetting-time values of the PMT coated-composite fabrics are evaluated all together (Figure 7), it can be seen that the hydrophilicity of the fabrics increased with the increment of the mass increase (%) of the composites. This observation can be related to the amounts of dopant anions incorporated into the PMT chains. Because with the increase of mass increases, more amounts of hydrophilic dopant ions should accompany the composite, resulting in a decrease of contact angle/wetting time.

### 3.3. Stability results

#### 3.3.1. Rubbing stability

**Figure 8:**
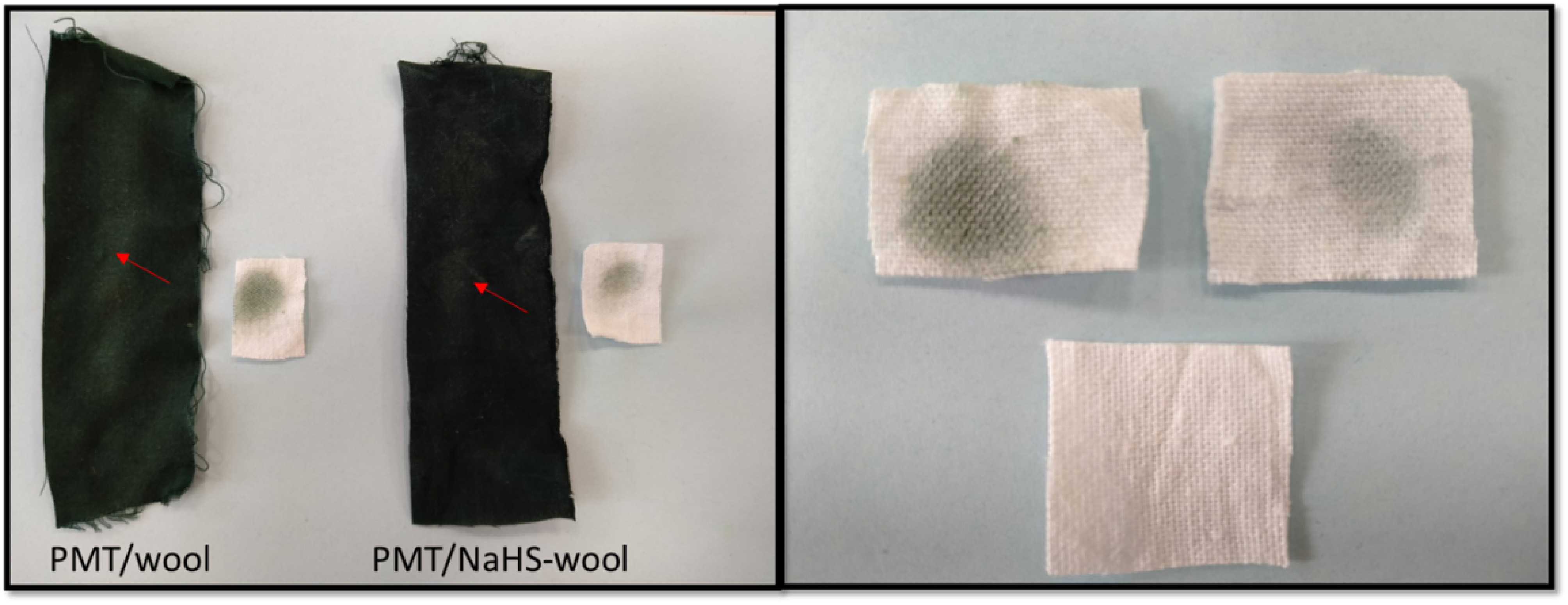
The optical images of the PMT/wool and PMT/NaHS-wool composites after the rubbing test.

To monitor the stability of the PMT coating on the wool surface against rubbing, the samples were subjected to a rubbing test with a white (bleached) cotton fabric for 10 times. The colour transferred to the cotton from PMT on the composites and change of surface resistivity of the composites were investigated, and the results, including the photographic images of the samples and ΔE colour distance values, are presented in Figure 8 and Table 4, respectively. As seen from the figure and table, the surface of bleached-cotton was significantly stained by the PMT coat of untreated-composite rather than that of the NaHS pretreated-wool (ΔE: 10.49), and this can be easily visualized through the darker shades of the cotton with a higher ΔE value of 41.50. The residual composites rubbed with the cotton fabrics had the different colour changes, and the untreated composite gave the impression of relatively more amounts of PMT coats that were removed from the surface through the rubbing compared to the pretreated-one. This observation can also be supported by the change of surface resistivity of the composites after rubbing. Since the surface resistivity of the untreated PMT/wool increased by 3.7 times after rubbing and reached to 1200 ±312 kΩ/cm^2^. This change was only by 1.3 times for PMT/NaHS-wool and measured as 93 ±26 kΩ/cm^2^ after rubbing. This finding shows that the NaHS pretreatment of wool significantly improves the stability of PMT coating on the surface and enables the firm adhesion of PMT onto the wool.

**Table 4:** The ΔE values of the samples calculated from their L a b values.

Sample	ΔL	Δa	Δb	ΔE
White bleached cotton^a^	0	0	0	0
Rubbed area PMT/wool	73	7	3	73.40
Rubbed area PMT/NaHS-wool	74	4	3	74.17
Stain on cotton PMT/wool	41	5	4	41.50
Stain on cotton PMT/NaHS-wool	10	1	3	10.49

^a^L a b values of white bleached cotton were taken as the reference, and the colour distance from this cotton was calculated.

#### 3.3.2. Washing stability

The stability of the mass increase (%) and surface resistivity of the PMT coated-wool composites against detergent washing were investigated, and the fabric samples were washed in the reference detergent (ECE B) containing solutions for 2 times. The results, including the changes in the fabric weights (%) and surface resistivity of the composites, are summarized in Table 5. After the first washing process of a PMT/wool composite, 6.3 % of weight loss was obtained due to the removal of uncoated PMT polymers and dopant anions present on the surface. Additively, the colour of the composite turned from dark green to dark blue as a result of dedoping that is a characteristic of polyanilines. Because the removal of dopant anions (here SO^-2^_4_) through washing causes a transformation of the emeraldine structure of polyaniline (green) to the nonconducting leucoemeraldine (blue). As a result, the surface resistivity of the composite took extremely high values over the limit of measurement of the multimetre (2000 MΩ/cm^2^), and thus, no further washing process could be applied to the composite. Since the low diffusion of hydrophilic PMT polymer onto the untreated hydrophobic wool internal sections (due to tight weaving), the dopants of PMT chains onto the wool may have easily removed by washing.

**Table 5:** 




After the first wash of the PMT/NaHS-wool composite, relatively a higher weight loss (7.5%) occurred compared to an untreated composite, and this may be arisen from the removal of aggregated PMT particles and dopant anions, due to heavy coating of PMT onto wool. Relatively, the surface resistivity of the composite increased by ~200 times and reached 17.6 MΩ/cm^2^. After the second wash, the colour of the composite started to change towards blue, and surface resistivity of the composite was still measured as 400 MΩ/cm^2^, even the 8.9% weight loss of the composite. This finding indicates that the PMT chains placed on the internal sections of the reduced-woven fabric may have enabled the conductivity. Because after the reduction pretreatment of wool with NaHS, the weaving tightness, namely, the pores of the woven relatively became enlarged compared to that of untreated one, and by the help of this, the PMT polymer could easily adhere to these sites through the seconder interactions such as H-bonding. Consequently, the mass increase, as well as conductivity of the composite, improved, and the composite showed relatively better stability behaviours.

### 3.4. EMSE results

**Figure 9:**
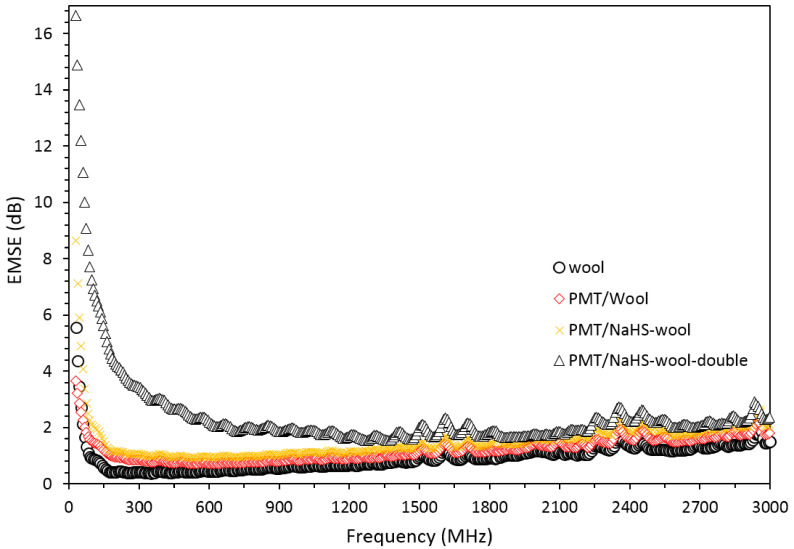
EMSE values of the wool, PMT/wool, and PMT/NaHS-wool composite that were coated once and twice within the frequency range of 30 MHz–3 GHz.

To examine the usability of PMT/wool composites in the field of EMI shielding, the EMSE measurements were performed in the frequency range of 30 MHz–3 GHz, and the results are presented in Figure 9. As seen from the figure, the untreated wool fabric had a minimal EMI shielding capability along with the tested frequency range with an average EMSE value of 0.9 dB. After the coating of this untreated wool with the PMT polymer, the EMSE value of the composite did not show a satisfactory improvement, and its average value stayed around 1.2 dB due to relatively low conductivity improvement compared to a pretreated composite. The EMSE value of the PMT/NaHS-wool composite was first measured as 8.6 dB at 30 MHz, it showed a decreased tendency with the increase of the frequency up to 300 MHz and then stayed almost constant at the average value of ~1.5 dB. After the second coating of this composite with the PMT polymer, the trend of the results did not remarkably change, but the results were relatively higher such as 16.6 dB at 30 MHz, 4 dB at 300 MHz and stayed average 2.5 dB between 300 MHz–3GHz as a possible result of increased conductivity of the composite.

When these EMSE values of the composites are evaluated together with the results of surface resistivity, one can expect that the increment of the conductivity of the PMT/NaHS-wool composite would result in a higher EMSE value because the EMI shielding phenomenon of a material significantly depends on the conductivity[24,34], and in this work, the surface resistivity (or conductivity) of the composite (22 kΩ/cm^2^) was improved by ca. 9 times compared to an untreated composite (203 kΩ/cm^2^). However, we could not reach such an expectation. The observation of such a low EMI shielding for double-coated composite should be related to the texture of the composite since the thickness of the material also has an improving effect on EMI shielding [34]. After the reduction pretreatment of wool with NaHS, the texture became weakened, the pores of the woven became enlarged, and thus, the vertical thickness of the fabric got thinner. Although the pretreated fabric was coated with PMT polymer twice, since the formation of PMT polymer possibly initiated from the internal sites of the woven and behaved like filling the composite, it might still be insufficient to increase its vertical thickness after reduction deterioration, and provide an expected high EMSE value to the composite.

## Conclusions

In this work, after the premodification of the wool with a reduction process, a homogenous and dense conductive PMT coating was ensured onto the wool surface through the in situ polymerization technique. The PMT/wool composite with the highest conductivity and mass increase (%) were obtained when the polymerization took place in 1N H_2_SO_4_ using NaHS reduced-wool fabric. The contact angle-wetting time measurements indicated the remarkable changes in the hydrophobic nature of the wool, with the contribution of hydrophilic PMT. Although the PMT/NaHS-wool composite showed relatively better EMI shielding performance rather than the untreated one, its EMSE value was still low due to the low thickness of the reduced wool. One of the most remarkable findings of the study was the rubbing and washing stability results, revealing the resistant and firm adhesion of PMT onto the reduced-wool surface compared to that of untreated one. This finding suggests that the surface of hydrophobic wool can be altered to impart the desired properties such as hydrophilicity.

## References

[1] (2019). Biofunctional wool using $\beta $-cyclodextrins as vehiculizer of citronella oil$. $ Process Biochem.

[2] (2013). Photodegradation of textile dye Rhodamine B over a novel biopolymer-metal complex wool-Pd/CdS photocatalysts under visible light irradiation$. $ Journal of Photochemistry and Photobiology B: Biology.

[3] (1972). A-a,. Polymer Journal.

[4] (2019). Graft polymerization onto wool fibre for improved functionality$. $ Progress in Organic Coatings.

[5] (1980). Grafting onto wool$. $ Polymer Bulletin.

[6] (2010). Grafting of chitosan as a biopolymer onto wool fabric using anhydride bridge and its antibacterial property$. $ Colloids and Surfaces B: Biointerfaces.

[7] (2016). Investigation of gelling behavior of thiolated chitosan in alkaline condition and its application in stent coating$. $ Carbohydrate Polymers.

[8] (2015). Aqueous ionic liquid solutions as alternatives for sulphide-free leather processing$. $ Green Chemistry.

[9] (1979). Process for producing reduced keratinous substances using urea or thiourea.

[10] (2016). Extracting keratin from wool by using l-cysteine$. $ Green Chemistry.

[11] (2003). Reduction mechanism of tioglycolic acid on keratin fibers using microspectrophotometry and FT-Raman spectroscopy$. $ Polymer.

[12] (2014). Extraction and characterization of keratin from bovine hoof: A potential material for biomedical applications$. $ SpringerPlus.

[13] (2019). Disulfide bond reconstruction: A novel approach for grafting of thiolated chitosan onto wool$. $ Carbohydrate Polymers.

[14] (2008). Action of thioglycolic acid and L-cysteine to disulfide cross-links in hair fibers during permanent waving treatment$. $ Sen'i Gakkaishi.

[15] (2019). Reactive keratin derivatives: A promising strategy for covalent binding to hair$. $ Journal of Colloid and Interface Science.

[16] (2007). Kaynak A. Improvement of adhesion of conductive polypyrrole coating on wool and polyester fabrics using atmospheric plasma treatment$. $ Synthetic Metals.

[17] (2019). Morphologically different silver particles decorated- conductive poly(o-anisidine)/wool fabric composites and investigation of catalytic activity in reduction of methylene blue$. $ Materials Chemistry and Physics.

[18] (2019). Deposition of Poly(o-anisidine) and Noble Ag Particles on Wool Fabric and The Evaluation of Its Performance as Catalyst in Dye Reduction. Journal of the Turkish Chemical Society Section A: Chemistry.

[19] (2005). A study on the electrical conductivity decay of polypyrrole coated wool textiles$. $ Polymer Degradation and Stability.

[20] (2005). Frictional and tensile properties of conducting polymer coated wool and alpaca fibers$. $ Fibers and Polymers.

[21] (2002). Characterization of conductive polypyrrole coated wool yarns$. $ Fibers and Polymers.

[22] (2008). Improving Electrical Performances of Wool Textiles: Synthesis of Conducting Polypyrrole on the Fiber Surface$. $ Textile Research Journal.

[23] (2016). Conductive poly (o-anisidine)/poly (ethylene terephthalate) nonwoven composite: Investigation of synthesis parameters and electromagnetic shielding effectiveness$. $ Journal of Industrial Textiles.

[24] (2017). Electromagnetic shielding of polypyrrole-sawdust composites: polypyrrole globules and nanotubes$. $ Cellulose.

[25] (1966). Separation of chemically unmodified histologica Lcomponents of keratin fibres and analyses of cuticles$. $ Nature.

[26] (1999). '{n}ska A. Application of Fourier-transform infrared and Raman spectroscopy to study degradation of the wool fiber keratin$. $ Journal of Molecular Structure.

[27] (2001). Study of vibrational spectra of polyaniline doped with sulfuric acid and phosphoric acid$. $ Applied Biochemistry and Biotechnology.

[28] (2011). The infrared spectroscopy of conducting polymer nanotubes (. IUPAC Technical Report).

[29] (1998). Vibrational Analysis of Polyaniline: A Model Compound Approach$. The Journal of Physical Chemistry B.

[30] (1998). Polyaniline: A polymer with many interesting intrinsic redox states$. $ Progress in Polymer Science.

[31] (2006). Evolution of Polyaniline Nanotubes: The Oxidation of Aniline in Water$. $ Journal of Physical Chemistry B.

[32] (2006). In-situ polymerized polyaniline films. Preparation in solutions of hydrochloric, sulfuric, or phosphoric acid$. $ Thin Solid Films.

[33] (1992). EMI shielding measurements of conductive polymer blends$. $ IEEE Transactions on Instrumentation and Measurement.

